# Providers' Perspectives on Provision of Family Planning to HIV-Positive Individuals in HIV Care in Nyanza Province, Kenya

**DOI:** 10.1155/2013/915923

**Published:** 2013-05-02

**Authors:** Sara J. Newmann, Kavita Mishra, Maricianah Onono, Elizabeth A. Bukusi, Craig R. Cohen, Olivia Gage, Rose Odeny, Katie D. Schwartz, Daniel Grossman

**Affiliations:** ^1^Department of Obstetrics, Gynecology and Reproductive Sciences, University of California, San Francisco, San Francisco General Hospital, 1001 Potrero Avenue, Ward 6D-14, San Francisco, CA 94110, USA; ^2^Department of Obstetrics and Gynecology, The Warren Alpert Medical School of Brown University, Box G-A1, Providence, RI 02912, USA; ^3^Centre for Microbiology Research, Kenya Medical Research Institute, P.O. Box 19464, Nairobi 00202, Kenya; ^4^University of North Carolina School of Medicine, 4030 Bondurant Hall, Campus Box 7000, Chapel Hill, NC 27599, USA; ^5^Ministry of Medical Services, Migori District Hospital, P.O. Box 202, Migori 40400, Kenya; ^6^Ibis Reproductive Health, 1330 Broadway, Suite 1100, Oakland, CA 94612, USA

## Abstract

*Objective*. To inform an intervention integrating family planning into HIV care, family planning (FP) knowledge, attitudes and practices, and perspectives on integrating FP into HIV care were assessed among healthcare providers in Nyanza Province, Kenya. *Methods*. Thirty-one mixed-method, structured interviews were conducted among a purposive sample of healthcare workers (HCWs) from 13 government HIV care facilities in Nyanza Province. Structured questions and case scenarios assessed contraceptive knowledge, training, and FP provision experience. Open-ended questions explored perspectives on integration. Data were analyzed descriptively and qualitatively. *Results*. Of the 31 HCWs interviewed, 45% reported previous FP training. Few providers thought long-acting methods were safe for HIV-positive women (19% viewed depot medroxyprogesterone acetate as safe and 36% viewed implants and intrauterine contraceptives as safe); fewer felt comfortable recommending them to HIV-positive women. Overall, providers supported HIV and family planning integration, yet several potential barriers were identified including misunderstandings about contraceptive safety, gendered power differentials relating to fertility decisions, staff shortages, lack of FP training, and contraceptive shortages. *Conclusions*. These findings suggest the importance of considering issues such as patient flow, provider burden, commodity supply, gender and cultural issues affecting FP use, and provider training in FP/HIV when designing integrated FP/HIV services in high HIV prevalence areas.

## 1. Introduction

Unmet need for contraception and unintended pregnancy are prevalent among the estimated 13 million HIV-positive women in sub-Saharan Africa [1–3]. Unintended pregnancies account for 14–58% of all births in countries where the burden of HIV is the greatest [4]. In South Africa, a recent cohort study of women attending antiretroviral (ART) clinics found that 62% of pregnancies were unintended [5], while a cross-sectional study of pregnant women obtaining services for prevention of mother-to-child transmission (PMTCT) reported that 84% of pregnancies were unintended [6]. In a cohort of Ugandan women starting ART, 17% became pregnant over the two-year follow-up period, despite 93% not wanting or planning pregnancy [1].

Prevention of unintended pregnancy among HIV-positive women is one of the World Health Organization's four cornerstones of preventing mother-to-child transmission of HIV (PMTCT) [7]. In many settings in sub-Saharan Africa, contraceptive services are provided in family planning (FP) clinics separate from clinics providing ART and related care for HIV-infected individuals [8]. Recognizing the structural barriers associated with this model of care, at least six international statements have recommended integrating family planning and HIV services in order to increase access among HIV-positive individuals to contraceptive counseling and services [2]. The US President's Emergency Plan for AIDS Relief (PEPFAR), the largest commitment from a single country to eradicate HIV, has identified decreasing unintended pregnancies among HIV-positive individuals as one of its four most essential strategies towards creating an AIDS-free generation [9]. Improved access to family planning among HIV-positive individuals is not only expected to decrease vertical transmission of HIV but also maternal morbidity and mortality, poor neonatal outcomes [10–12], and various other health and societal cost outcomes related to unintended pregnancies and vertical transmission of HIV [4, 13–15].

 The Kenyan government has been working towards implementing integrated family planning and HIV services throughout the country for several years. In 2007 the Reproductive Health (RH) HIV Integration Technical working group, a Ministry of Health-led task force cochaired by representatives from the Division of Reproductive Health and the National AIDS and STI Control Program, was created and has been critical in advancing family planning/HIV integration efforts throughout the country. In 2009 this multidisciplinary group developed a National RH and HIV and AIDS Integration Strategy [16] which provided a framework for the integration of RH and HIV services. Recently, in 2012, the RH-HIV Integration Technical working group created a blueprint for national implementation of integrated family planning and HIV services [17]. In order to add to the evidence needed to support such national integration efforts in Kenya and in other sub-Saharan African countries, members of this taskforce from the Kenya Medical Research Institute, University of California, San Francisco, and Ibis Reproductive Health initiated a cluster randomized trial evaluating the impact of FP/HIV integration on contraceptive prevalence and unintended pregnancy [18].

 Health care providers are at the forefront of defining, offering, and/or modifying integrated HIV and family planning services, yet little research has been done in Kenya and elsewhere exploring the perspectives of health care providers with regard to integrated reproductive health and HIV services [19–23]. In preparation for the cluster randomized trial we sought to explore the viewpoints of health care providers working in public sector HIV care and treatment clinics in rural, western Kenya (Nyanza Province) with respect to providing family planning services within HIV care settings.

In Nyanza, the overall HIV prevalence is 14 percent, the highest in the country and double the level of the next highest provinces—Nairobi and Western—at 7 percent each. Gender differences in HIV prevalence exist here; it is estimated that 16 percent of women and 11.4 percent of men are HIV-positive [15]. The total fertility rate in Nyanza is 5.4 and the contraceptive prevalence rate for modern methods is 33%, despite the fact that 75% of married men and women in Kenya age 15–49 years report a desire to delay fertility for at least two years or cease childbearing all together. Among married women of reproductive age, approximately 18% report using depot medroxyprogesterone acetate, 6% female sterilization, 3% oral contraceptives, 4% condoms, and less than 2% intrauterine or subdermal methods [15]. Unmet need for contraception among HIV-positive women in Kenya appears even higher than that of the general population [8]. 

Our study aimed to assess providers' knowledge and attitudes regarding family planning provision for HIV-positive individuals, as well as their perceptions of benefits and barriers to integrating contraceptive provision into HIV care. This preliminary work was conducted to explore whether providers felt integrated FP/HIV services were feasible and sensible within HIV clinics and to inform the design of the intervention component of a cluster randomized controlled trial (RCT) evaluating the impact of integrating family planning into HIV care. 

## 2. Methods

We conducted a mixed-method study between November 2007 and October 2008 at thirteen government-run HIV care and treatment clinics “patient support centers” in the Migori, Rongo, and Suba districts of Nyanza Province, Kenya. The study sites selected were supported by Family AIDS Care and Education Services (FACES), a collaboration between the University of California San Francisco (UCSF) and the Kenyan Medical Research Institute (KEMRI). FACES provides training, clinical mentorship, and logistical support for public sector HIV care and treatment clinics in these districts in western Kenya [24].

The 13 study sites included public sector dispensaries, health centers, and subdistrict and district hospitals, 11 of which were sites to be included in the RCT and two of which were district hospitals that had already begun to integrate family planning services into HIV care. All study sites provided comprehensive HIV care to their clients, including ART. The study participants included 31 paid healthcare workers at these sites, over half of whom had a clinical diploma (nurses and clinical officers), while the others were counselors or clinic assistants who had regular contact with patients. The decision to include lay healthcare workers, also called community clinic health assistants (CCHAs), was purposeful. In a response to a shortage in healthcare workers, FACES staff has implemented “task-shifting” [25] and has trained lay healthcare workers to conduct the majority of counseling regarding medication adherence, side effects, and HIV prevention at these health facilities. CCHAs were part of the healthcare workforce at all study sites and it was anticipated that they would be involved in patient screening and education for family planning services after integration was implemented.

The study participants were selected through purposive sampling, with the goal of interviewing 30–40 providers where the intended family planning and HIV care intervention would occur. At each study site, an interviewer approached healthcare workers present that day and invited them to participate in the study. The interviewer explained that the purpose of the study was to learn about HIV providers thoughts on and experience with providing family planning to HIV-positive men and women as well as their thoughts on if and how family planning should be provided for this population. The interviewer asked if they would be willing to participate in an approximately one-hour interview. All 31 providers approached for the study agreed to participate. Two to three providers were interviewed from each site. The number of study participants is equivalent to approximately one-third of the healthcare workforce at the thirteen sites. Participants were offered a book voucher worth 350 Kenyan shillings (about USD $4) after the interview.

 The study instrument consisted of two parts: a structured, quantitative survey and an open-ended interview. Structured questions and case scenarios were used to assess contraceptive knowledge, training, and provision experience, while open-ended questions provided the opportunity to discuss opinions about the reproductive intentions of their clients and about the possibility of integrating family planning into HIV care. The case scenarios were included to explore providers' knowledge regarding the safety of different contraceptive methods with respect to HIV and whether or not their practice patterns would differ based on fertility intentions, age, and other sociodemographic factors. Providers were presented with three clinical scenarios in which HIV-positive women with differing parities, ages, comorbidities, and fertility intentions desired contraception. In each scenario all contraceptive methods are considered to be clinically safe for use in the context of HIV infection and disease, according to WHO medical eligibility for contraception guidelines [26]. They were asked method-by-method (which included condoms, oral contraceptives, depot medroxyprogesterone acetate, and intrauterine, subdermal, and permanent contraception) if each method was safe for the patient and if they would recommend that method to the patient. The quantitative portion of the study instrument was modeled after an evaluation of a family planning and antiretroviral therapy integration pilot in Mbale, Uganda [27]. The instrument was reviewed by and piloted with nonstudy participant Kenyan HIV healthcare providers and subsequently revised prior to finalization and data collection.

The qualitative portion of the instrument was informed by questions used in an open-ended interview guide from a study of providers' perspectives on the reproductive intentions of HIV-positive individuals in Cape Town, South Africa [28]. Domains included providers' views on childbearing among and current provision of family planning for HIV-positive individuals and thoughts on integrating family planning into HIV care. The interviews were conducted in English in a private room in the healthcare facility and lasted approximately one hour. With participant consent, the interviews were audio-recorded and subsequently transcribed. No identifying information was recorded in the audio recordings or interview notes.

All quantitative data were analyzed using Stata 9.2 (College Station, TX, USA). Frequencies were generated and appropriate comparisons were made using Fisher's exact and Chi-square tests. Qualitative data were analyzed using a grounded theory approach [29]. Initial thematic categories were drawn from the literature and the interview transcripts and then subcategorized once the full range of themes and patterns was developed. Participant responses to questions were coded manually by SN and KM. Trends and crosscutting themes were identified and further explored during the final analysis. Any coding discrepancies were resolved through discussion and consensus.

This study was approved by the Ethical Review Committee at KEMRI and the Committee of Human Research at UCSF. All participants gave written informed consent prior to study participation.

## 3. Results

### 3.1. Demographics and Clinical Context

Of the 31 providers, 18 were clinicians (clinical officers or nurses) and 13 were HIV/VCT (voluntary counseling and testing) counselors, community clinic health assistants, or health worker volunteers who had regular contact with patients in HIV care. The median number of years as a healthcare provider was 3 (range: 1–26) and of HIV care experience was 1 (range: 1–11).

 Twenty-four of the respondents reported that condoms were available at their health facility in general, not necessarily at the HIV clinic, and 17 reported the availability of at least one non-barrier contraceptive method, such as oral contraceptive pills, depot medroxyprogesterone acetate (DMPA), or subdermal or intra-uterine contraception (IUC) (Table 1). Eight respondents reported that at least one long-acting reversible contraception method (subdermal or intra-uterine contraception) was available at their site, and only two of these eight reported availability of subdermal implants. Half of the providers reported working at a site where there was a provider trained in IUC insertion.

Two-thirds (19) of the respondents reported that they desired additional family planning training. Fourteen providers reported receiving some family planning training outside of their initial schooling during the previous two years. The respondents with recent family planning training were more likely to be working at a larger facility, for example, subdistrict or district hospital, than at a health center or dispensary (*p* = 0.03).

### 3.2. Main Themes

#### 3.2.1. Theme I: Choice-Based Perspective regarding Fertility in the Context of HIV

In order to explore potential biases among providers with respect to HIV and pregnancy, participants were asked about their personal views regarding an HIV-positive women becoming pregnant and whether they think it is appropriate for HIV-positive men and women to bear children. All providers stated that an HIV-positive person has a right to have a child. However, their personal views about HIV-positive people bearing children differed depending on client sex. In general, providers viewed HIV-positive women as having the right to bear children. However, they qualified this choice by stressing that an HIV-positive woman's health should be optimized for pregnancy and if she is not in good physical condition, they would not recommend conception. Their concern appeared to lie mainly with the potential maternal health risks associated with HIV disease and pregnancy and less commonly with the risk of maternal-child transmission. One nurse said,

*“Generally, her health should be good. Her CD4 should be high; her viral load should be low…because (pregnancy) can do her more harm if her health is not optimized…”*

A woman's choice of pregnancy was further qualified by her marital status and by the number of children she already had. Most providers commented that the woman should be married if she planned to get pregnant and she should not have many children already.

A few providers acknowledged that they are rarely faced with an HIV-positive female client who desired pregnancy and that thinking about their reactions to such a client was hypothetical. They said they usually encountered HIV-positive women who were already pregnant or wanted to prevent pregnancy, not women who desired pregnancy.

When asked about their views on HIV-positive men fathering children, providers tended to think about the man in the context of his marriage, family, and community, rather than his health. Providers spoke more frequently about the importance of partner agreement regarding having a child. One nurse said,

* “I'll just tell him…to bring the wife, to talk to the wife, and do consultation with each other and see if they have one conclusion.”*

In addition to concerns about family size, providers expressed concerns about the familial and social impacts of an HIV-positive man fathering a child, especially in the situation of limited resources. They stated concerns about men being able to financially support their families and to continue to work and be productive members of society. 

 Providers almost unanimously expressed views about family planning that reflected a focus on reproductive choice and family health. One community clinic health assistant said,

*“Family planning to me means…planning the number of children you want to have and (when) you want to have them…there is a way of giving birth to the number of children you can take care of.”*

The survey portion of the interview additionally revealed a rights-based perspective. Twenty-seven (87%) respondents said that HIV-positive people were free to have sex if they wanted. These views did not differ if the respondent was a clinician or nonclinician.

When asked their views on appropriate family planning counseling for HIV-positive men and women, most providers said it was important for women to consult their partners prior to using family planning; however, this was not expressed for men. For men, they often stressed the importance of dual protection, while for women this was less commonly mentioned. When asked about what they would recommend if an HIV-positive man or woman knew they did not want to have any more children, most providers recommended female sterilization for both hypothetical male and female clients. Few providers mentioned male sterilization.

#### 3.2.2. Theme 2: Views regarding Hypothetical Integration of HIV Care and Family Planning: Facilitators and Barriers

When they were asked about integrating family planning and HIV services, several barriers and facilitators were mentioned. Most providers were supportive of the idea and felt it would improve patient care. One community clinic health assistant said,

*“I think it would be good to integrate services so that clients are not being transferred from one place to the other, they might even disappear on the way…”*

A nurse said,

*“When the patients come to the clinic all these services can be given there instead of seeing the patient partially and then referring the patient for family planning services…” *

Several providers reflected on the current situation in which they felt HIV-positive women were given few family planning options. They reflected on male resistance to condom use and how often providers had nothing to give women other than condoms, but if her partner refused to use this method, then they really were not providing family planning. They also expressed concerns about staffing issues, method supply, space, and training limiting their ability to provide family planning. One nurse said,

*“If all we have are condoms, we may tell a woman to use a condom…And then the partner does not want to use the condom…so when she comes back we tell her can we give you more condoms? She says no because the husband does not want to use…And if she wants another family planning method that is not within the facility…then she will not get the service. And also if she needs the service that is inside (the facility) and the provider is not there, she will not get it…”*

One clinical officer said,

*“We do not have enough drugs. You can counsel somebody…but how are you going to (provide family planning) if you do not have the pills? If you do not have private space? Or if you cannot insert an IUC or do a tubal ligation?”*

Providers also voiced concerns about method-related fears among clients. Most spoke about women's concerns about side effects such as irregular bleeding. When asked what family planning myths exist in their communities, providers mentioned misperceptions about contraceptive safety and potential teratogenicity. Among various myths mentioned, providers said that intrauterine contraception is believed to sometimes travel to the brain or the heart, and some people believe that men *“will be disturbed psychologically” *after vasectomy. Providers felt that societal myths about family planning served as significant barriers to use regardless of HIV status and efforts to overcome these misperceptions should be incorporated into integration plans.

Despite their concerns about the logistics of integration and family planning misperceptions, there were several social factors that they thought might facilitate the success of integration. Providers talked about stigma, both toward people with HIV and toward people using family planning, as factors that would be better addressed through integration. With regard to HIV stigma, providers felt that having family planning services available within the HIV clinic would protect HIV-positive clients from unnecessary discrimination and feelings of shame due to discomfort with disclosing one's HIV status outside of the HIV clinic. One community clinic health assistant said,

*“If a client is HIV positive and needs family planning methods…she should get it within (the HIV clinic)…not be referred to another place and be given that service outside…because she may go there and she may not say that she is HIV positive..”*

Several providers also mentioned stigma against women attending family planning clinics and the assumption that women who do so are promiscuous. One nurse said,

*“Family planning uptake is still low because…the community feels those people who go for family planning…have many sexual partners so one would wonder why she has to go for family planning if she only has one sexual partner with whom she can easily organize.”*

Participants felt that having family planning available at the HIV clinic would minimize the impact of HIV-related and family planning-related stigma and increase contraceptive use. Providers felt the HIV clinic was a good place for family planning because they *“understand their (patients') problems better (than the staff at the family planning clinic).”*

 Most providers mentioned roles that men play in family planning. Two salient and pervasive themes emerged. Providers discussed how currently men seemed to play a prohibitive role against using family planning, but how incorporating men more effectively into family planning decision-making through integrated services might be beneficial. Providers expressed views that even in their absence men dictate women's choices of family planning and influence the way in which providers counsel women. They talked about how most women choose DMPA because it is a covert form of contraception. One nurse said,

*“In our community, the Luos, most…use Depo because…they know their husbands will not be able to realize very fast.” Providers also felt “we can improve (family planning) by including the males.” *

They felt that incorporating men into family planning counseling and decision-making could decrease clandestine behavior and increase awareness of partners' needs, thereby creating a situation that is more conducive to family planning continuation. They felt integration of family planning into HIV care would facilitate inclusion of men since men are generally more comfortable coming to the HIV clinic, where there are other men or where they might be a patient themselves, than accompanying their partner to the family planning clinic, almost exclusively attended by women.

#### 3.2.3. Theme 3: Knowledge and Providing Patterns among Providers regarding Contraceptive Safety in HIV-Positive Individuals

**Figure 1:**
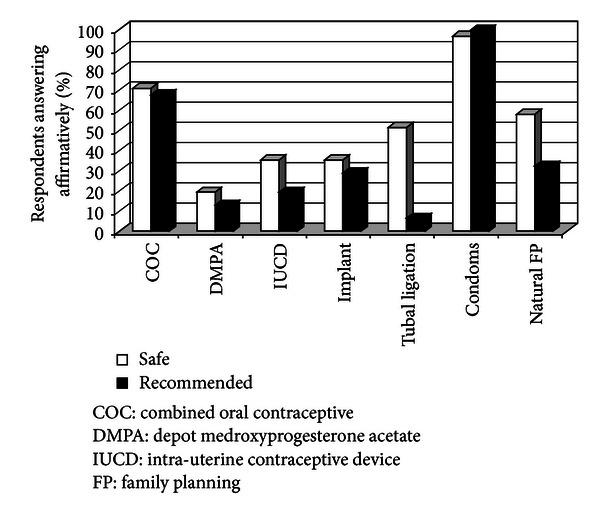
Contraceptive safety knowledge and recommendation of contraceptive methods among HIV care providers in Nyanza Province, Kenya (*N* = 31).

Correct knowledge regarding the safety of family planning methods and what providers would recommend to HIV-positive clients was low (see Figure 1). Despite the safety of DMPA, IUC, and contraceptive implants for use by the women in the clinical scenarios, only 19.4%, 35.5%, and 35.5% of respondents, respectively, thought these methods were safe. Clinicians were significantly more likely than nonclinicians to correctly report the safety of combined oral contraceptives and tubal ligation (*p* = 0.02 and *p* = 0.01, respectively). Tubal ligation was least recommended, with only 6.5% of respondents reporting that they would recommend it. Condoms were deemed the safest and the most recommended method. Only four participants (13%) erroneously thought that condoms were the only family planning method an HIV-positive person can use.

With the exception of the differences in family planning safety knowledge, differences in responses according to the respondent's role in the clinic (nurse, clinical officer, or CCHA) were not observed. Overall response themes were quite uniform, and there were few outliers with respect to the themes described above.

## 4. Discussion

Similar to other studies from sub-Saharan Africa [26, 27], we found that the majority of providers viewed pregnancy as a basic right for people living with HIV. However, they reported in their experience that HIV-positive women more commonly wanted to limit or end childbearing rather than conceive. They stated that HIV-positive people should have access to all contraceptives. Providers were enthusiastic about integrating family planning into HIV care and felt integration could improve access to contraception and reduce stigma related to both family planning and HIV. However, we found that the providers interviewed had extremely limited knowledge and uncertainty about the safety of contraceptive methods, hormonal and nonhormonal, and whether or not to recommend contraception to people living with HIV. Our study findings portray the enthusiasm and hypothetical acceptability among HIV providers for FP/HIV integration. Our findings also reveal the dire need to comprehensively educate HIV providers about the safety of FP methods with respect to HIV and to increase their ability to incorporate sensitivity to complex gendered power differentials that influence contraceptive choice and use into their counseling. These data informed the development of the FP/HIV intervention in Nyanza, Kenya, used in the cluster randomized trial which is now being used as a model to guide national integration efforts in Kenya and will be useful in similar resource-poor settings in sub-Saharan Africa. 

Providers identified several sociocultural barriers with respect to contraceptive uptake. They spoke about the need for partner approval before using family planning and how as a result of this gendered power dynamic most women in Kenya choose to use DMPA [15], the most easily concealable contraceptive. These findings of male partner influence on contraceptive choice and use, while uncovered in the context of a study on FP/HIV integration, are issues that have long been recognized in the literature and among practitioners as obstacles to FP use among women in the general population. Previous research in Kenya demonstrated that the strongest predictors of female contraception use were male fertility preference [30] and partner disapproval which predicted use of less effective family planning methods or, more commonly, none at all [31–33]. Providers felt that involving men in fertility decision-making could potentially help promote women's and their families' health. 

We found that providers were more likely to counsel men to use dual protection and to tell women to consult their partners before using family planning, implying that perhaps there was a perception that men are at greater risk of transmitting HIV than women are and/or that men have more agency in protecting themselves from acquiring HIV or impregnating a woman than women do with respect to HIV and pregnancy prevention. The majority (approximately 80%) of patients seen at the HIV clinics participating in the cluster RCT are married, with approximately a fifth of them reporting being in polygamous relationships [18]. Given these statistics, integrating family planning into HIV care could have a major impact on contraceptive use among married men and women since it enables the counseling and provision of family planning to be done in an environment where men are expected to be, different from the maternal-child health clinics generally attended only by women. However, issues related to stigma associated with coming to an HIV clinic in general must be recognized.

Male involvement in family planning needs to be implemented in a way that promotes joint and equitable decision-making about family planning and does not reinforce gendered norms about men as decision makers. In order for increased male involvement in family planning to occur in a way that is safe and constructive for women, gender-sensitive approaches to training of HIV care providers with respect to HIV and pregnancy prevention need to be used. As part of the family planning integration intervention we implemented couples family planning counseling at integrated sites. However, couples counseling will only be appropriate for a portion of HIV-positive individuals who present for HIV care as it requires agreement on behalf of both partners to engage in joint counseling and assumes a level of acceptance that joint decision-making is important and feasible. In order to engage a broader population of HIV-positive men and women in family planning, further development, implementation, and evaluation of interventions that promote gender-equitable reproductive health behavior and decision-making are important in order to create family planning and HIV integrated services that are effective and will decrease unintended pregnancies among HIV-positive individuals.

As it has been found in many countries [34] regardless of HIV infection, providers identified widespread community and personal misperceptions regarding side effects and safety of contraception to be a barrier to contraceptive provision and uptake. Accurate training is needed on the provider level with respect to family planning in order for HIV providers to feel comfortable counseling and providing FP methods to HIV-positive patients. Education is also needed on the community level, in and outside of the clinics, in order to help dispel many of the socially perpetuated myths that exist about contraceptive use. As a result of these interviews, FP educational curricula and job aids were developed to be used in the FP/HIV intervention in order to dispel contraceptive misperceptions and to train community clinic health assistants and certified health care providers to provide FP group education, counseling, and methods [35]. These tools are now being used nationally to scale up the implementation of FP/HIV. 

Participants expressed concerns about lack of staff, space, and methods as barriers to integrating family planning into HIV care; similar concerns have been voiced by providers elsewhere in Kenya and Uganda [26, 36]. The HIV epidemic has fueled a major crisis in the healthcare workforce, especially in sub-Saharan Africa. The rapid expansion of people needing treatment for HIV-related illness has overwhelmed healthcare systems globally [37]. The WHO has responded to this shortage in the healthcare workforce with recommendations for “task-shifting,” [25]. Task-shifting has been found to be cost-effective in several African countries [38] and has been successfully implemented for ART provision in urban regions of Kenya [33]. Given the growing patient volume and health needs of HIV-positive individuals, implementing task- shifting within integrated services appears essential [34].

Most research on family planning and HIV has not focused on providers but rather on the physiologic effects of hormonal contraception and antiretroviral therapies [39–41] and on the unmet need for contraception and fertility desires among HIV-positive clients [1, 42–45]. We found that knowledge about the safety and appropriate use of family planning methods among HIV-positive individuals was limited as it has also been found in Uganda [26]. We also found that providers were more likely to counsel men to use dual protection and to tell women to consult their partners before using family planning. Further research into the ways in which preconceived ideas regarding gender roles and gendered power differences influence HIV care providers is essential in order to create effective family planning training programs for providers.

This study has several limitations. The most important limitation is that the providers were asked hypothetical questions about integrated services. None of the participants had significant experience providing integrated services and thus provided theoretical responses. Multiple issues, such as logistic and financial factors, that are important to the provision of integrated services may not have been thoroughly discussed and responses were not reflective of actual experiences with integration. The study was conducted in a limited number of facilities, each of which had a small number of providers. Additionally, it was conducted only in clinics that are currently supported by FACES and would be participating in the FP/HIV integration cluster RCT. Although the RCT had not begun and providers were not aware of the plans for the RCT, it is possible that providers in these clinics have different views on family planning compared to providers in non-FACES-supported clinics. While the findings are likely representative of these districts, they may not be representative of providers in Kenya as a whole. Another difficulty relates to the tragic postelection violence that occurred in Kenya in late 2007 and early 2008. During this time all data collection ceased for nine months. Despite discontinuous data collection, we observed no important differences in participants' responses before and after this period.

 HIV-positive women in sub-Saharan Africa have a significant unmet need for contraception [1–3, 8, 42, 43], and unintended pregnancy among HIV-positive women is prevalent [1, 4–6, 44, 45]. Although more data on clinical outcomes are needed, integration of family planning into HIV care will likely improve access to family planning for HIV-positive individuals and reduce stigma. Given the growing evidence base supporting a focus on family planning as an integral component of decreasing vertical transmission and eradicating HIV, as well as recognition of the reproductive rights of HIV-positive individuals, resources must be devoted to address the challenges identified by HIV health care providers regarding training, staffing, clinic space, commodity distribution, and gender-based fertility decision-making identified here.

## Figures and Tables

**Table 1:** Demographics and clinical context of HIV care providers (*N* = 31).

Clinical experience	
Age in years (median, range)	33 (30–35)
Clinicians (clinical officers or nurses)	18 (58%)
Community clinic health assistants	13 (42%)
Number of years worked as provider (median, range)	3 (1–26)
Number of years worked in HIV care (median, range)	1 (1–11)
Clinical site for family planning availability	
Worked at sub-district or district hospital	15 (48%)
Worked at site where condoms were available	24 (80%)
Worked at site where at least one nonbarrier method was available [OCP's, DMPA, implants, or IUC]	17 (57%)
Worked at site where implants or IUC was available	8 (26%)
Family planning training	
Received training in family planning outside of school, during the past two years	14 (45%)
Desires additional family planning training	19 (61%)

OCP: oral contraceptive pills.

DMPA: depot medroxyprogesterone acetate.

IUC: intrauterine contraception.
